# A New Thermal Controlling Material with Positive Temperature Coefficient for Body Warming: Preparation and Characterization

**DOI:** 10.3390/ma12111758

**Published:** 2019-05-30

**Authors:** Jing Li, Chang Chang, Xue Li, Yang Li, Guoqiang Guan

**Affiliations:** 1 School of Chemistry and Chemical Engineering, South China University of Technology, Guangzhou, Guangdong 510640, China; cechangc@mail.scut.edu.cn (C.C.); qlianlixue@sina.com (X.L.); 2 SCUT-Zhuhai Institute of Modern Industrial Innovation, Zhuhai, Guangdong 519175, China; 3 School of Mechanical and Automotive Engineering, South China University of Technology, Guangzhou, Guangdong 510640, China; meyangli@scut.edu.cn

**Keywords:** positive temperature coefficient materials, Curie temperature, thermal control, body warming, preparation

## Abstract

Positive temperature coefficient (PTC) materials have many applications in self-regulating heating; however, they are generally used in the high temperature range, and their use in the room temperature range have rarely been reported. A new kind of PTC material with the Curie temperature of 37 °C was proposed in this study. The material was prepared by adding 6wt % carbon black (CB) and 5wt % dioctyl phthalate (DOP) into the copolymer of ethylene vinyl acetate (EVA) and lauric acid (LA) (1:3). The results showed that it had an exceptionally high PTC intensity of 5.5 and rationally low electrical resistivity of 600 Ω·cm. at room temperature. And this PTC material also shows a preferable repeatability in the PTC intensity after the thermal cycle of 4th. Moreover, the PTC material shows a great potential application in warming the human body in a cold environment.

## 1. Introduction

Thermal control has been widely used to keep the human body warm in a cold environment, like outer space and polar regions. A material with the positive temperature coefficient (PTC) effect has shown a bright prospective in thermal control due to its low cost, light weight, flexibility, and self-regulation.

The positive temperature coefficient materials are a kind of temperature-sensitive materials, whose electrical resistivity sharply increase with a raised temperature over the threshold, i.e., Curie temperature [1]. Traditionally, PTC materials are widely used in the electrical industry such as self-limiting fuses [2] and sensor elements [3,4], as well as heating elements for adaptive temperature controls such as self-regulating heating cables [5,6,7]. Using PTC composites for adaptive control of temperature reduces the weight or volume of the thermal control system, whose temperature control electronic components are eliminated, and combining PTC composites with proportional-integral-derivative (PID) components can improve the accuracy of thermal control system [8,9].

Recently, the Curie temperatures of most existing PTC materials are mostly in the range of 50–400 °C [10,11,12]. However, PTC composite materials with high Curie temperatures are not suitable for body warming applications such as temperature control equipment for biological insulation and infant incubators. Few studies have been found in the literature on PTC materials with the Curie point at room temperature, because it is difficult to obtain the two preferable properties of high PTC intensity and low room temperature resistivity simultaneously [13]. Cheng et al. prepared a series of composites which have the suitable electrical resistivity of 10^3^ Ω·cm at the Curie temperature of 34 °C and a PTC intensity of about 3 [14]. A PTC intensity of 2 was reported by using first-order phase transitions in percolated composite materials with a Curie point of about 18 °C [15,16]. Unlike the work to prepare the PTC materials with the suitable electrical resistivity but low intensity, Cheng et al. presented that the PTC material has the preferred intensity of 5.0 with resistivity of about 13600 Ω·cm [17]. A higher resistivity of 10^5^ Ω·cm was found in studies on an amorphous PS/CSPE-MWCNT composites [18]. The high resistivity leads to a higher excited voltage, which may pose a potential risk of electric shock.

In this work, the PTC material with a Curie point of 37 °C, which presented high intensity and low electrical resistivity at room temperature was prepared. The dose influence of raw materials on the room temperature resistivity, PTC intensity, and Curie temperature were focused. Additionally, the repeatability of the optimized composition was also investigated. The PTC microstructural model was established to explain the increasing room temperature resistivity due to thermal cycles. The relationship between the Curie temperature and the melting point of PTC composite materials was investigated using differential scanning calorimetry (DSC). The relationship between the distribution of conductive particles and the properties of the PTC composites was determined using scanning electron microscopy (SEM).

## 2. Materials and Experiment

### 2.1. Materials 

The ethylene vinyl acetate (EVA, Commodity grade: VA910, VA mass fraction: 28%, density: 0.950 g/cm3, melt index: 25 g/10 min at 190 °C/2.16 kg) was supplied by Korea Han Hua Technology Co. Ltd., and the lauric acid (LA) was provided by Tianjin Fortune Chemical Reagent Co. Ltd. The acetylene carbon black (CB, 20–40 nm) was supplied by Fushun Carbon Co. Ltd., and the dioctyl phthalate (DOP), acetone, and xylene were provided by Shanghai Run Chemical Reagent Co. Ltd.

### 2.2. Preparation of PTC Composites

#### 2.2.1. Pretreatment of Acetylene Black

Acetylene CB was dipped in acetone for 24 h to remove organic substances that may have been adsorbed on the surface of the acetylene black. After filtration, the acetone was removed by vacuum distillation. Then, the acetylene CB was heated to 100 °C and held for 120 min to remove volatile substances on the acetylene CB surface.

#### 2.2.2. Preparation of the Composites

The EVA powder and LA particles were added to a 250 ml beaker using the raw material ratios listed in Table 1, and then poured into approximately 50 ml xylene. Under the condition of mechanical agitation and heating to 60 °C, corresponding amounts of CB and DOP were added according to the ratios in Table 1 until the mixture was completely dissolved. The solution was stirred for 10 min. The solution was ultrasonicated at a frequency of 37.1 Hz and temperature of 55 °C for 1 h. Next, the mixed liquid was cast on 50 mm by 50 mm gauze, and the xylene solvent was volatilized using an electric blower drying box at 60 °C and maintained at a constant temperature for 1 h. Then, the drying box temperature was changed to 100 °C and maintained at a constant temperature for 2 h. The product was cooled to room temperature while in the electric blower drying box; next, the temperature was increased to 40 °C at 2 °C min^-1^ and the temperature was held constant for 2 h. Then, the product was cooled to room temperature.

### 2.3. Methods and Instruments

In order to measure the electric performance of the PTC materials, two copper electrodes were placed on both ends of the sample, and a thermocouple was attached to the center. The sample was put in the high-low temperature test chamber (BPH-060A, Shanghai Yiheng Instrument and Meter Co. Ltd., China), with the temperature ranged from 20 °C to 50 °C at a heating rate of 1 °C min^−1^, and measured the resistance (UT61E, Uni-Trend Technology Co., Ltd, Dongguan, China) with different temperatures (TM-902C, Zhengzhou Heng Sen instrument and Meter Co., Ltd., China). After testing, the resistivity (ρ) could be calculated according to the formula: ρ = RS/L, where R is the test resistance of the sample, L is the length of the sample, and S is the cross-sectional area of the sample.

The melting behavior of the PTC composites was studied using a DSC (DSC-Q20, NETZSCH-Ger Tebau GmbH, Shanghai, China) which operated from 10 °C to 65 °C at a heating rate of 5 °Cmin^−1^ in a nitrogen atmosphere. A SEM (SU8200, Hitachi High-Technologies Co. Ltd., Shanghai, China) was used to study the microstructures of the PTC composites sectional structure, and the images were taken with a SU8200 microscope.

## 3. Results and Discussion

### 3.1. Optimal PTC Effect at Different Material Proportions 

**Figure 1:**
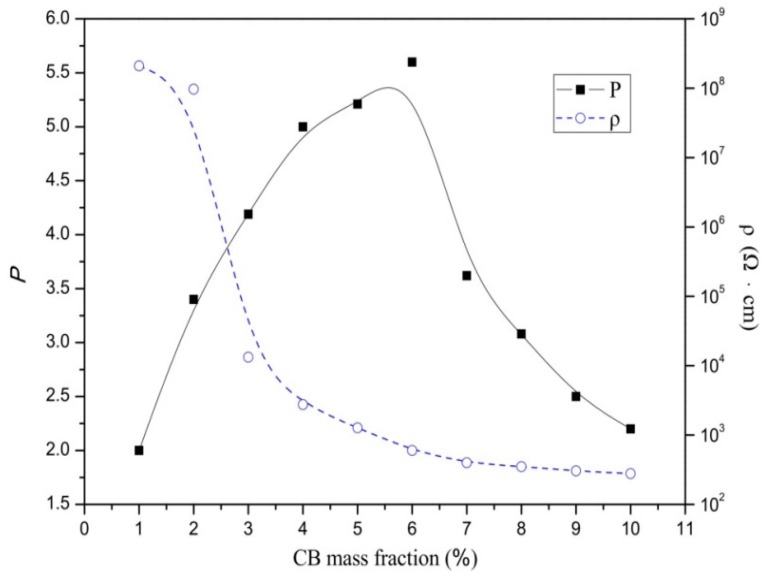
Effect of carbon black (CB) content on electrical resistivity and positive temperature coefficient (PTC) intensity of composites 1–10.

Firstly, we investigated the effect of CB content on material properties. The resistivity for each sample of composites 1–10 at 25 °C varied at different CB mass fractions, as shown by the dashed line in Figure 1. It can be observed from the curve that the percolation threshold was approximately 6%. The room temperature (25 °C) resistivity decreases slowly when the CB content is lower than 2% and the resistivity remains approximately 10^8^ Ω·cm, which indicates that the composite is an insulator. When the CB content increases to between 2% and 6%, the room temperature resistivity of the composites sharply decreases from 10^8^ Ω·cm to 10^2^ Ω·cm with increasing CB content, and the material becomes a semiconductor. When the CB content exceeds 6%, the room temperature resistivity of the material barely changes.

The PTC intensity (P) of PTC material is defined as the logarithmic ratio of the maximal electrical resistivity (ρ_max_) to the minimal one (ρ_min_) at the investigated temperature range, i.e., the effect of CB mass fraction on the P are also shown in Figure 1 (solid line). To improve the PTC intensity of the composite, the CB content should be selected near the percolation zone and at the lower side of the room temperature resistivity. If the CB content is too high and the temperature exceeds the Curie temperature, then temperature changes do not provide enough energy to damage the conductive network, which leads to less variation in the resistivity. On the other hand, if the CB content is too low, the initial resistivity at room temperature is very large even when the CB particles are fully separated within the matrix; however, there is insufficient room for mutation and the final PTC intensity is still low. The P–ω (CB) curve in Figure 1 validates the above analysis. Consequently, when the CB mass fraction was 6%, the material had a relatively low ρ of approximately 600 Ω·cm, and a high P of 5.5, which is the optimal CB content for thermal control.

**Figure 2:**
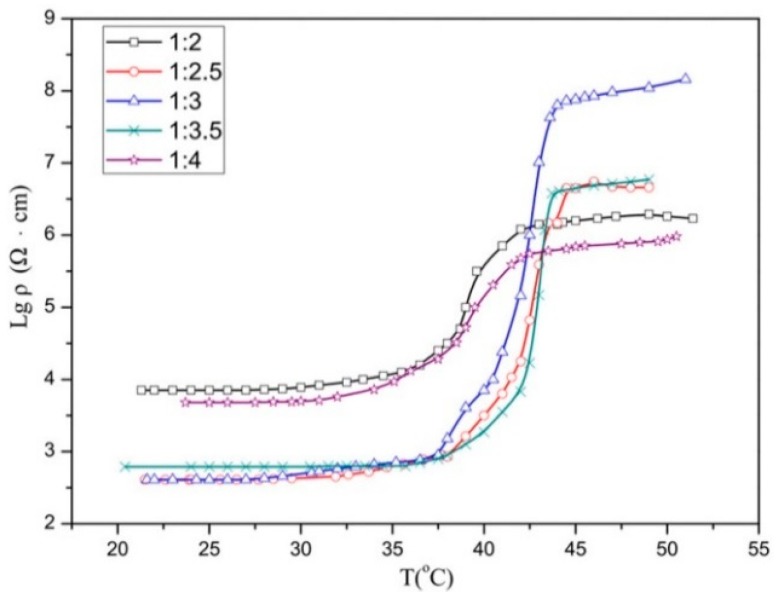
Logarithmic resistivity–temperature curves at different ethylene vinyl acetate (EVA)/ lauric acid (LA) ratios for composites 6A–6E.

After defining the optimum CB content, the CB content was held constant and the ratio of EVA to LA was varied while the sum of both was held constant at 94%. The resistivity–temperature curves of samples 6A–6E are shown in Figure 2. To characterize the results in terms of intuitionistic logic, the room temperature resistivity, and PTC intensity shown in Figure 2 are summarized in Table 2.

The logarithmic resistivity–temperature curves of 6A and 6E had narrow span in the direction of the vertical axis; in other words, as the temperature increases, the increases in the resistivity were significantly lower than those of the other three lines. Similarly, the increases in resistivity of 6B and 6D were also lower than that of 6C. As presented in Table 2, the P of 6C was larger than those of other four values. Composite 6C had the highest PTC intensity and the lowest room temperature resistivity compared to other composites. Consequently, Figure 2 and Table 2 show that at EVA/LA ratio of 1:3, the material had a relatively low 25 °C resistivity, and relatively high PTC intensity.

**Figure 3:**
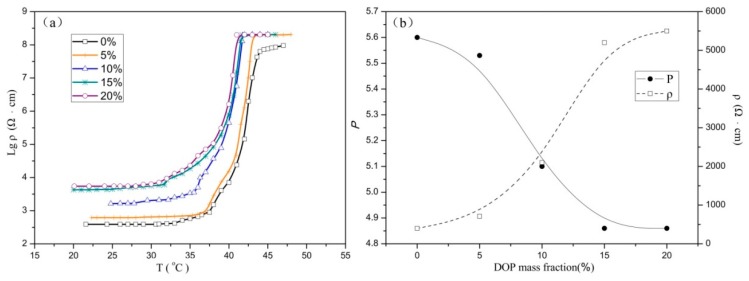
Effect of dioctyl phthalate (DOP) content on material properties. (**a**) is logarithmic resistivity–temperature curves of composites A6–E6; (**b**) is the change curves of PTC intensity and room temperature resistivity at different DOP contents.

To lower the Curie temperature, a plasticizer was added to the composites. The resistivity–temperature curves of samples A6–E6 are shown in Figure 3a. All five samples have the same CB mass fraction of 6%, the same EVA/LA mass proportions of 1:3, and the DOP content was varied from 0% to 20%. The Curie temperature of composites A6–E6 are listed in the Table 3. It shows that the Curie temperature decreased with increasing DOP content. As a plasticizer, the DOP can weaken the secondary bond between polymer molecules, weaken the force between molecular chains, increase the mobility of molecular chains, improve the flowability of resin processing, and reduce the crystallinity. In the composite, the crystals making up the matrix material probably became more refined as the DOP content increased. When the temperature increased, the fine crystallites were more likely to melt and become smaller, and a volume change of the crystal probably occurred in the conductive channel. For this reason, the temperature required to destroy the conduction path decreased and the Curie temperature decreased. 

The effect of different DOP mass fractions (ω) on room temperature ρ and P of the composites are shown in Figure 3b. From the ρ–ω curves, it is apparent that as the DOP mass fractions increased, the ρ increased. From the P–ω curve, it is shown that as the DOP mass fraction increased, the PTC intensity decreased gradually. If the mass fraction of DOP is small, the probability of DOP crystal refinement occurring is minimal. Typically, when the temperature exceeds the Curie temperature, the degree of crystalline transformation is large, and the resistivity of the material changes significantly, resulting in high PTC intensity. At increasing DOP content, the internal crystal shape of the composite is gradually refined, and when the temperature exceeds the Curie temperature, less change occurs in the crystalline phases, thereby reducing the maximum resistivity. 

In addition, the room temperature resistivity also increased with increasing DOP content; therefore, the PTC intensity decreased with increasing DOP content. Combining the results shown in Figure 3, the optimum DOP content is 5%, the room temperature resistivity of the composite is low, and the PTC intensity is high.

### 3.2. Repeatability of the PTC Effect for Composite 6C

**Figure 4:**
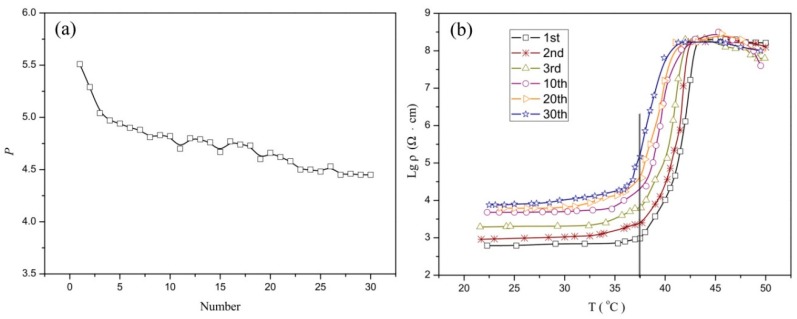
Repeatability of the PTC effect. (**a**) is PTC intensity versus thermal cycles; (**b**) is logarithmic resistivity–temperature curves of composites in different repetitions.

In order to research the repeatability of the PTC behavior, taking the optimal sample 6C as an example, 30 thermal cycles from 20 °C to 50 °C are repeated, PTC intensity versus thermal cycle number curve is given in the Figure 4a.

As the curve shows, the PTC intensity of composite revealed a decrease trend with the increasing thermal cycles and the change decreased after 4 cycles, but it still retained excellent PTC characters. The specific logarithmic resistivity–temperature curves are shown in Figure 4b. The high temperature (40 °C to 50 °C) resistivity is relatively stable, while the room temperature (25 °C) resistivity is gradually increased. The increase in room temperature resistivity is the main cause of the decrease in PTC intensity. With respect to the increase in room temperature resistivity, it may result from the incompletely recoverable changes in composite structure, volume, aggregation state, and distribution of conductive particles, once a thermal cycle occurs. It may be an inherent phenomenon, which can be found in other studies [19,20,21], and a more clear explanation could be found in Section 3.4. As shown in Figure 4b, the Curie temperature of sample 6C did not change with the number of thermal cycles, because of that the Curie temperature depends on the phase transform temperature of the composite, which is an inherent property of the material and is not affected by thermal cycles.

### 3.3. Curie Temperatures of PTC Composites

**Figure 5:**
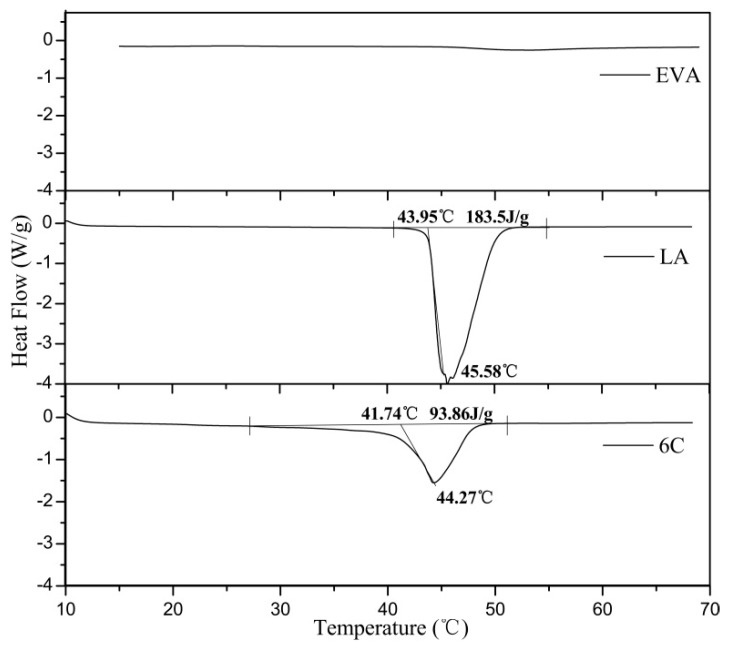
DSC thermogram of EVA, LA, and composite 6C.

DSC measurement of the composites was carried out to verify the accuracy of the Curie point. Figure 5 shows the DSC thermogram of EVA, LA, and PTC composite 6C. A comparison of the three DSC data indicates that the curve of EVA is smooth without phase change from 15 °C to 65 °C, and there is one sharp endotherm evident in both DSC curves of LA and composite 6C representing the transition from crystalline to amorphous phase. The endothermic peak temperatures and Curie points of composites 6A–6E on their DSC curves are presented in Table 4.

The peak temperature of composites 6C–6E is near the peak temperature of LA, and the Curie temperature for each composite was approximately 37 °C, which is slightly below their peak temperatures. As mentioned previously, CB mass fractions had no influence on the Curie temperature such that the Curie temperature of the PTC composites was determined by the phase change temperature, and LA was the decisive phase change material of the PTC composites.

### 3.4. Microstructural and Model Analysis

**Figure 6:**
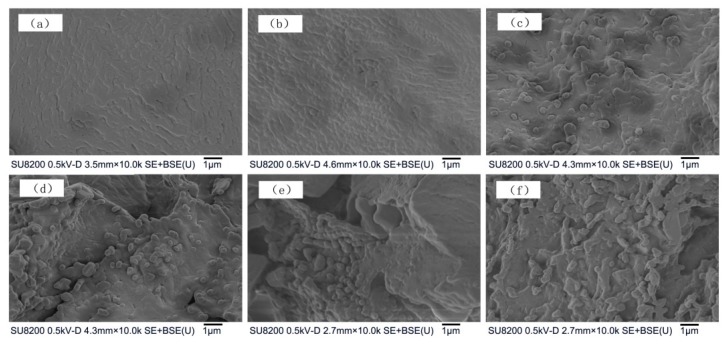
SEM micrographs of PTC composites with CB weight percent of (**a**) 1%, (**b**) 2%, (**c**) 6%, (**d**) 6%, (**e**) 7%, and (**f**) 10%.

Figure 6 shows SEM micrographs of the composites with varying amounts of CB. The results in Figure 6 can be explained with the help of Figure 1. By comparing the conductive composites and different CB contents, it is apparent that each has a different morphology and CB distribution. Figure 6a,b show the conductive composites at CB contents of 1% and 2%, respectively. The morphology of the surface is smooth, and the network of conductive CB is not continuous. In other words, the distribution is discrete, and the filler is distributed in an “Island-Island” form. The room temperature resistivity was high as the formation of the conductive network was not continuous, therefore, the PTC intensity of the composite 1 and 2 was low.

Figure 6c,d show the SEM micrograph of PTC composites with CB contents of 6%, where the conductive CB filler is uniformly distributed in the matrix formed by EVA/LA. In addition, the distance between conductive regions is suitable, which may form some complete conductive passages, thus reducing the room temperature resistivity. Increasing the temperature above the Curie point can separate the CB regions and destroy the conductive network, resulting in higher resistivity change, and thus higher PTC intensity.

Figure 6e,f show SEM micrographs of conductive composites with CB contents of 7% and 10%, respectively. These figures clearly show that the conductive CB filler formed a perfect conductive network in the composite, and agglomeration is apparent in some localized areas due to excessive CB. Although agglomerates reduce the overall resistivity of the composite materials at room temperature, the change in the crystal shape is not enough to separate the CB particles and destroy the conductivity at the Curie temperature point, resulting in low resistivity and a small change in the resistivity, and hence low PTC intensity. 

**Figure 7:**
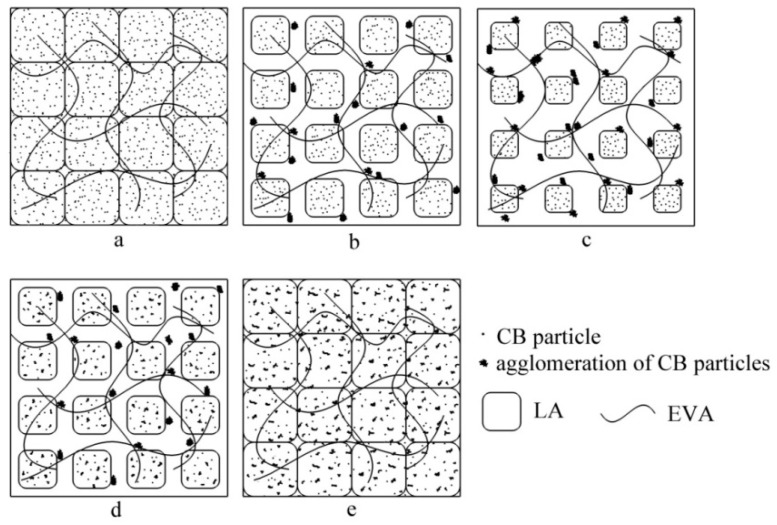
Microstructural transition model (**a**–**e**) of PTC materials during a thermal cycle.

To explain clearly the cause of PTC phenomenon, microstructural transition model of PTC materials during a thermal cycle is schematically illustrated in Figure 7.

From the results, the PTC phenomenon may be explained by changes in the microstructure of the composite. When the temperature was below the Curie point, according to the model in Figure 7a, CB particles were arranged randomly in the matrix due to strong dispersion during the preparation. As the content of CB particles reached a certain value, the conductive particles make contact with each other and formed a complete conductive network with good conductivity and low resistivity.

As the temperature increased above the phase transition temperature, the structure of the composite changed dramatically. As shown in Figure 7b,c, crystal volume became smaller and CB particles started to form CB agglomerates, and the distance between conductive regions increased gradually, which may destroy the conductive network, resulting in significant increase in the resistivity.

From Figure 7d,e, when temperature decreased, crystal formed again and CB agglomerates would be broken up into smaller CB agglomerates, but they were not the CB particles which were dispersed well as shown in Figure 7a. Of course, there is a limit to the agglomeration of carbon black, so the increase in room temperature resistivity after multiple thermal cycles was reduced. Accordingly, with the microstructural transition model, it is easy to understand the increasing room temperature resistivity due to thermal cycles in the previous Section 3.2.

## 4. Conclusions

In this study, we prepared a novel heating material with PTC effect, whose Curie temperature was 37 °C. Especially, by adding CB conductive particles to EVA and LA, along with a DOP plasticizer, PTC intensity of 5.5, and room temperature resistivity of 600 Ω·cm was achieved. As verified by DSC, LA as a low melting point organic acid crystal dominated the Curie temperature of the material. The CB content had influence on the room temperature resistivity and PTC intensity of the composites. Furthermore, in the repeatability test, the PTC intensity of the material weakened initially but tended to be stable eventually, which can be explained by using the microstructural transition model we established.

## Figures and Tables

**Table 1:** Weight fractions (ω) of raw materials for PTC composites.

No.	ω (CB)	ω (EVA)/ω (LA)	ω (DOP)	No.	ω (CB)	ω (EVA)/ω (LA)	ω (DOP)
1	1%	1:3	–	6A	6%	1:4	–
2	2%	1:3	–	6B	6%	1:3.5	–
3	3%	1:3	–	6C	6%	1:3	–
4	4%	1:3	–	6D	6%	1:2.5	–
5	5%	1:3	–	6E	6%	1:2	–
6	6%	1:3	–	A6	6%	1:3	–
7	7%	1:3	–	B6	6%	1:3	5%
8	8%	1:3	–	C6	6%	1:3	10%
9	9%	1:3	–	D6	6%	1:3	15%
10	10%	1:3	–	E6	6%	1:3	20%

**Table 2:** Electrical resistivity and PTC intensity of composites 6A–6E.

Composites No.	6A	6B	6C	6D	6E
EVA/LA ratio	1:4	1:3.5	1:3	1:2.5	1:2
25 °C resistivity (Ω·cm)	4800	610	390	410	7070
PTC intensity P	2.3	3.92	5.6	4.13	2.44

**Table 3:** Curie temperature of composites 6A–6E.

Composites No.	A6	B6	C6	D6	E6
DOP content	0	5	10	15	20
Curie temperature (°C)	38	37	35	33	32

**Table 4:** Endothermic peak temperatures and Curie points of composites 6A–6E.

Sample Name or No.	LA	6A	6B	6C	6D	6E
Peak temperature (°C)	44.0	41.9	42.9	41.7	41.1	40.3
Resistivity jump point (°C)	–	37.1	37.5	37.5	37.7	37
